# Switchable Tri-Functional Terahertz Metamaterial Integrated with Vanadium Dioxide and Photosensitive Silicon

**DOI:** 10.3390/nano15110835

**Published:** 2025-05-30

**Authors:** Gui Jin, Ying Zhu, Haorui Yang, Bin Tang

**Affiliations:** 1 Department of Electronic Information and Electronic Engineering, Xiangnan University, Chenzhou 423000, China; jingui0531@xnu.edu.cn; 2 School of Microelectronics, Changzhou University, Changzhou 213164, China; s24060809031@smail.cczu.edu.cn

**Keywords:** metamaterial, perfect absorption, polarization conversion, asymmetric transmission

## Abstract

This work presents a theoretical and numerical investigation of a switchable tri-functional terahertz metamaterial incorporating vanadium dioxide (VO_2_) and photosensitive silicon. The selective absorption, broadband linear-to-linear polarization conversion, and dual-band asymmetric transmission (AT) can be realized by utilizing the phase transition characteristic of VO_2_. When VO_2_ behaves as a metal, the proposed metamaterial functions as a selective perfect absorber for *x*-polarized waves at 2.84 THz, while exhibiting near-zero absorption for *y*-polarized waves. When VO_2_ is in its insulating state, the proposed metamaterial acts as a linear polarization converter, achieving a polarization conversion ratio exceeding 99% within the frequency range of 1.07 to 4.29 THz. Meanwhile, a dual-band AT effect can be simultaneously realized associated with the broadband near-perfect polarization conversion. Furthermore, the polarization conversion efficiency and AT can be actively modulated by adjusting the conductivity of the photosensitive silicon, offering a novel approach for realizing multifunctional terahertz devices.

## 1. Introduction

Terahertz wave is an attractive frequency band due to its strong penetration and non-ionization [1], which plays an important role in wireless communication [2], biomedicine [3], and security detection [4]. Metamaterial, an artificially manufactured material arranged in periodic arrays [5,6], can exhibit some novel electromagnetic behavior in the terahertz band [7,8], which has attracted tremendous interest for its wide range of applications, such as perfect absorption [9], beam control [10], optical imaging [11], electromagnetic cloaking [12], polarization conversion [13], and slow light effect [14], etc. In particular, metamaterial absorbers have a relatively flexible design and smaller dimensions compared to conventional absorbers. In recent years, metamaterial absorbers have been extensively investigated across the electromagnetic spectrum, with diverse applications ranging from the microwave to visible frequency regimes [15,16,17,18], since Landy [19] first demonstrated the metamaterial perfect absorber. In addition, asymmetric transmission (AT), whose properties are similar to optical diode, represents the difference in transmission of electromagnetic waves in opposite directions [20,21]. Interestingly, associated with AT effect, polarization conversion can usually be efficiently achieved by utilizing metamaterials [22], which facilitate the transformation of electromagnetic wave polarization states. For example, Zhou et al. designed two types of ultra-broadband infrared absorbers with small period and super thin thickness [23]. Tian et al. proposed an all-dielectric germanium metamaterial absorber in the near-infrared region [24]. Zhao et al. presented an innovative metamaterial structure which can achieve ultra-broadband and high-efficiency linear polarization conversion as well as AT effect in the microwave region [25]. However, conventional terahertz metamaterials typically operate at fixed frequencies and offer single functionality after fabrication, underscoring the pressing need for dynamically reconfigurable and multifunctional designs to meet the demands of diverse applications [26,27]. The development of dynamically tunable, multifunctional metamaterial devices is highly desirable to address the diverse application needs of terahertz technology. To achieve the controllable multi-functionality of the metamaterials device, one feasible design scheme is to combine metamaterials with functional materials, such as phase change materials [28,29], two-dimensional materials [30,31], semiconductors [32,33], and so on. Among these materials, vanadium dioxide (VO_2_) is a representative phase change material that undergoes a dramatic transition from an insulating to a metallic state at around 340 K [34,35]. Since the phase change process is reversible and can be accomplished in a few picoseconds, some VO_2_-integrated multi-functional metamaterials have been reported to achieve reconfigurable functions [36,37,38]. For instance, Tang’s group reported a VO_2_-graphene hybrid metamaterial exhibiting electrically tunable multifunctional responses in the terahertz regime, which can reversibly switch between AT and two different polarization conversions [39]. Wei et al. reported a multifunctional VO_2_-based metamaterial with a multilayer structure with a switchable broadband absorption and a polarization conversion in the terahertz region [40]. In particular, photosensitive silicon [41] is a promising functional material whose conductivity can be modulated by applying a pump light source with different intensities [41,42,43,44]. Nevertheless, to our knowledge, limited research exists on achieving switchable functionality between perfect selective absorption, AT effect, and polarization conversion within terahertz hybrid metasystems incorporating photosensitive silicon.

In this paper, we propose and numerically validate a dynamically reconfigurable tri-functional metamaterial, leveraging VO_2_ and photoactive silicon for terahertz-wave manipulation. By the phase transition of VO_2_, the switching among single-band selective absorption, double-band near-perfect AT and broadband perfect polarization conversion can be achieved. Specifically, when VO_2_ is in a metallic state, the designed metamaterial structure exhibits selective absorption characteristics, which can achieve perfect absorption for the *x*-polarized wave in 2.84 THz and near-zero absorption for the *y*-polarized wave. When VO_2_ is in the insulating state, dual-band AT effect with an efficiency~0.98 can be obtained at 2.72 THz and 3.84 THz. Meanwhile, broadband polarization conversion can be accomplished with polarization conversion ratios exceeding of 0.99 for the *x*-polarized wave in the range of 1.07–4.29 THz. Moreover, the polarization conversion performance and the OFF/ON state of AT effect can be tuned by changing the intensities of pump light acting on the photosensitive silicon. The switchable and tunable metamaterial may offer new possibilities for the design of terahertz multi-functional nanophotonic devices.

## 2. Structure and Methods

**Figure 1:**
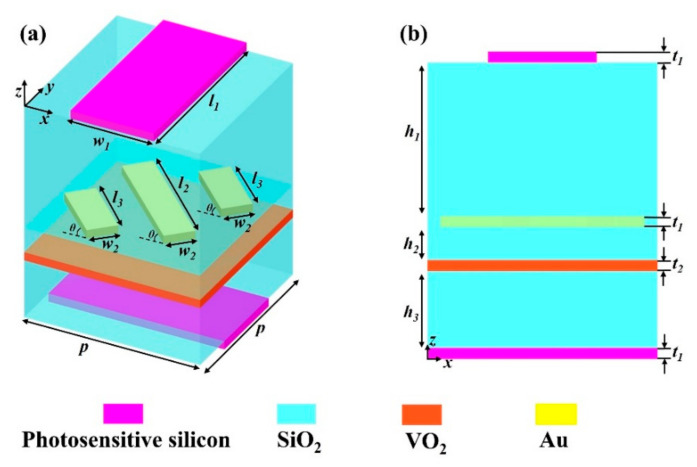
(**a**) Unit-cell architecture of the proposed metamaterial. (**b**) Cross-section view along the *x*-*z* plane.

Figure 1a illustrates the three-dimensional unit cell of the proposed metamaterial. From top to bottom, the metamaterial structure comprises a photosensitive silicon strip, dielectric spacer, Au grating, VO_2_ film, dielectric spacer, and photosensitive silicon strip. The Au grating is rotated at *θ* of 45° relative to the *x*-axis and embedded in the first dielectric spacer. The top and bottom photosensitive silicon strips are of the same size, with an angle of 90° relative to each other, which are placed in the center of the top or bottom layer. Figure 1b shows the side view of the unit cell in the *x-z* plane. The geometric parameters of the structure, optimized through numerical simulations, are provided below: *p* = 21 μm, *w*_1_ = 14.5 μm, *w*_2_ = 4.2 μm, *l*_1_ = 21 μm, *l*_2_ = 21 μm, *l*_3_ = 10.5 μm, *t*_1_ = 0.4 μm, *t*_2_ = 1 μm, *h*_1_ = 9.6 μm, *h*_2_ = 3 μm, *h*_3_ = 6 μm. In fact, the proposed design aligns with established fabrication technologies, ensuring practical feasibility. The fabrication process begins with a thoroughly cleaned silicon substrate. A bottom photosensitive silicon strip is first deposited onto the substrate using plasma-enhanced chemical vapor deposition (PECVD). Next, a dielectric layer is applied, followed by the formation of a VO_2_ layer via either pulsed laser deposition (PLD) or magnetron sputtering. A subsequent dielectric layer is then deposited, and a gold (Au) layer is added atop this structure. To pattern the Au layer, a polymethyl methacrylate (PMMA) resist is spin-coated onto its surface, and the desired grating structure is defined using ion beam milling (IBM). After removing the residual PMMA through a lift-off process, an additional dielectric layer is deposited. Finally, a top photosensitive silicon strip is formed on the dielectric layer via PECVD, completing the device architecture.

The electromagnetic responses of the metamaterials were calculated using the three-dimensional finite-difference time-domain (FDTD) method. In the numerical simulations, periodic boundary conditions were applied along the *x*-, *y*-directions, while perfectly matched layers were implemented in the *z*-direction to absorb outgoing waves and minimize reflections. To demonstrate the AT effect of the metamaterial, we define the plane wave propagating along the negative direction of *z*-axis as forward direction and the positive direction of *z*-axis as backward direction. In addition, the SiO_2_ was set as a lossless material with a refractive index of 1.96 in the terahertz band [45]. The conductivity of the photosensitive silicon can be tuned by varying the power of pump light source in a wide range, reaching the order of 10^6^ S/m [41]. The effective permittivity of Au and VO_2_ at terahertz band can be described by the Drude model [39]:

(1) $$\varepsilon_{Au}(\omega )=1-\frac{\omega_{p}^{2}}{\omega^{2}+i\gamma_{Au}\omega },$$

(2) $$\varepsilon_{VO_{2}}(\omega )=\varepsilon_{\infty }-\frac{\omega_{p}^{2}(\sigma )}{\omega^{2}+i\gamma_{VO_{2}}\omega },$$

where *ω* is the incident angular frequency, the plasma frequency of Au is *ω_p_* (Au) = 1.37 × 10^16^ rad/s, and *γ_Au_* = 4.08 × 10^13^ rad/s is the collision frequency of Au. The permittivity at an infinite frequency is *ε_∞_* = 12, and $\gamma_{{VO}_{2}}$ = 5.75 × 10^13^ rad/s is the collision frequency of VO_2_. The conductivity *σ* of VO_2_ is related to its plasma frequency ω*_p_* through the following expression:

(3) $$\omega_{p}^{2}(\sigma )=\frac{\sigma }{\sigma_{0}}\omega_{p}^{2}(\sigma_{0}),$$

where σ_0_ = 3 × 10^5^ S/m and *ω*_p_(*σ*_0_) = 1.4 × 10^15^ rad/s.

The incident and transmitted electric fields are related through the Jones matrix as follows [46]:

(4) $$\left(\begin{array}{c} T_{x}\\ T_{y} \end{array}\right)=\left(\begin{array}{cc} t_{xx}^{f} & t_{xy}^{f}\\ t_{yx}^{f} & t_{yy}^{f} \end{array}\right)\left(\begin{array}{c} I_{x}\\ I_{y} \end{array}\right)=T_{lin}^{f}\left(\begin{array}{c} I_{x}\\ I_{y} \end{array}\right),$$

where *I_x_* and *I_y_* denote the complex amplitudes of the incident wave components in the *x*- and *y*-directions, while *T_x_*, and *T_y_* represent those of the transmissive wave. The terms *t_xx_* and *t_yy_* are the co-polarization transmission coefficients, and *t_yx_* and *t_xy_* are the cross-polarization transmission coefficients. The superscript “*f*” indicates the forward incidence of the electromagnetic wave, and the subscript “*lin*” indicates the linearly polarized wave. According to the Lorentz reciprocal theorem, the backward-propagating wave using the Jones matrix can be expressed as:

(5) $$T_{lin}^{b}=\left(\begin{array}{cc} t_{xx}^{b} & t_{xy}^{b}\\ t_{yx}^{b} & t_{yy}^{b} \end{array}\right)=\left(\begin{array}{cc} t_{xx}^{f} & -t_{xy}^{f}\\ -t_{yx}^{f} & t_{yy}^{f} \end{array}\right),$$

where the superscript “*b*” denotes the wave incident from the backward direction. In addition, the degree of AT can be expressed by the parameter Δ*_lin_* as:

(6) $$\Delta_{lin}(x)=T_{x}^{f}-T_{x}^{b}={\left|t_{xx}^{f}\right|}^{2}+{\left|t_{yx}^{f}\right|}^{2}-{\left|t_{xx}^{b}\right|}^{2}-{\left|t_{xx}^{b}\right|}^{2},$$

(7) $$\Delta_{lin}(y)=T_{y}^{f}-T_{y}^{b}={\left|t_{yy}^{f}\right|}^{2}+{\left|t_{xy}^{f}\right|}^{2}-{\left|t_{yy}^{b}\right|}^{2}-{\left|t_{xy}^{b}\right|}^{2}=-\Delta_{lin}(x),$$

where Δ*_lin_*(*x*) and Δ*_lin_*(*y*) represent the asymmetric transmission (AT) parameters for *x*- and *y*-polarized waves under the forward illumination, respectively.

To evaluate the operating performance of polarization conversion, the polarization conversion ratio (PCR) can be defined as follows:

(8) $$\left\{\begin{array}{l} {\mathrm{PCR}}_{x}=\frac{\left|t_{yx}^{2}\right|}{\left|t_{yx}^{2}\right|+\left|t_{xx}^{2}\right|}=\frac{T_{yx}}{T_{yx}+T_{xx}}\\ {\mathrm{PCR}}_{y}=\frac{\left|t_{xy}^{2}\right|}{\left|t_{xy}^{2}\right|+\left|t_{yy}^{2}\right|}=\frac{T_{xy}}{T_{xy}+T_{yy}} \end{array}\right.,$$

where PCR*_x_*, PCR*_y_* denote the cross-polarization conversion ratios for *x*- and *y*-polarized incident waves, respectively.

## 3. Results and Discussion

**Figure 2:**
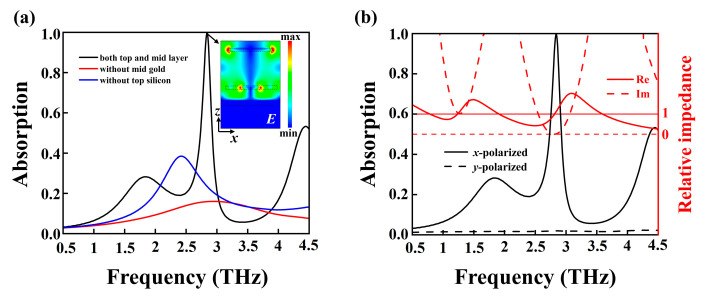
(**a**) Absorption of various metamaterial architectures. The black curve represents the structure incorporating Au gratings and a photosensitive silicon layer, the blue curve is without a top photosensitive silicon layer and the red curve is without middle Au gratings. The inset shows the electric field distribution of the metamaterial on the *x*-*z* plane at 2.84 THz. (**b**) The left axis represents the absorption spectra under forward illumination for *x*-polarized (solid black line) and *y*-polarized (dashed black line). The right axis is the relative impedance of the metamaterial: real part (solid red line) and imaginary part (dashed red line).

When VO_2_ is in the metallic state, the VO_2_ layer effectively blocks the transmission of the electromagnetic wave, so the absorption (*A*) of the metamaterial can be simplified as *A* = 1 − *R*, where “*R*” represents the reflection of the wave. It can be found from Figure 2a that there is a low absorption of the metamaterial without the middle Au gratings indicated by the red line. In contrast, there appears to be a resonant peak with absorption efficiencies less than 0.4 for the metamaterial without top photosensitive silicon layer as indicated by the blue line. Notably, the proposed metamaterial demonstrates narrowband perfect absorption of electromagnetic waves at the resonant frequency of 2.84 THz. To elucidate the underlying physical mechanism, we analyze the electric field (*E*) distribution in the *x*–*z* plane at this frequency, as illustrated in the inset of Figure 2a. One can see from the inset that the localized surface plasmon polaritons are excited in the top photosensitive silicon and Au grating layers when *x*-polarized waves are incident on the metamaterial. Here, the photoactive silicon’s conductivity is set to 5 × 10^6^ S/m based on prior experimental characterization. As a result, the electromagnetic interaction between the two adjacent layers leads to high absorption. Figure 2b depicts the absorption characteristics under a linearly polarized forward-irradiation wave with different polarizations. It can be observed that the absorption reaches nearly 100% at 2.84 THz for *x*-polarized waves, whereas the proposed metamaterial exhibits near-zero absorption for *y*-polarized waves. Thus, the proposed metamaterial functions as a polarization-selective near-perfect absorber for linearly polarized terahertz waves.

The perfect absorption behavior of the metamaterial can be interpreted through the impedance matching theory [47]. Based on Fresnel formalism, the reflection coefficient *r* is expressed as:

(9) $$r=\frac{Z-Z_{0}}{Z+Z_{0}},$$

where *Z* denotes the metamaterial impedance and *Z*_0_ represents the free-space impedance. Therefore, the absorption of metamaterials can be expressed as:

(10) $$A=1-R=1+{\left|\frac{Z-Z_{0}}{Z+Z_{0}}\right|}^{2}=1-{\left|\frac{Z_{r}-1}{Z_{r}+1}\right|}^{2},$$

in which *Zr* = *Z*/*Z*_0_ is the relative impedance. Equation (10) demonstrates that peak absorption occurs at the impedance-matched condition (Z = Z_0_). The complex relative impedance, including both real and imaginary components, can be obtained through S-parameter inversion:

(11) $$Z_{r}=\pm \sqrt{\frac{{\left(1+S_{11}(\omega )\right)}^{2}-S_{21}^{2}(\omega )}{{\left(1-S_{11}(\omega )\right)}^{2}-S_{21}^{2}(\omega )}}.$$

As indicated on the right axis of Figure 2b, the relative impedance at 2.84 THz approximates Re(*Z_r_*) ≈ 1 and Im(*Z_r_*) ≈ 0, achieving near-perfect impedance matching with free space (*Z*_0_), which directly correlates with the observed 100% absorption peak.

**Figure 3:**
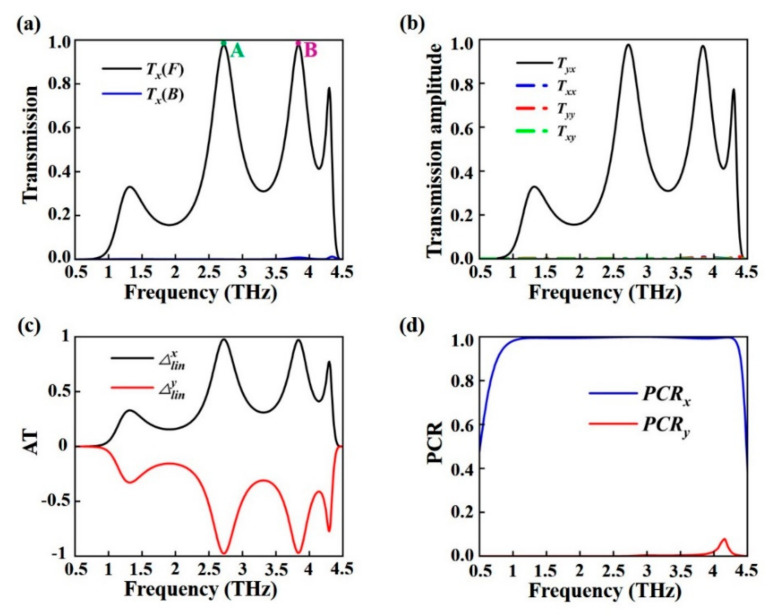
(**a**) Total transmission for *x*-polarized waves under forward and backward incidence. (**b**) Transmission amplitudes of linearly polarized waves. (**c**) AT and (**d**) PCR spectra for both polarizations.

When VO_2_ is in its insulating state, the VO_2_ film behaves as a transmissive medium, so the electromagnetic waves can pass through the structure. In this case, the photoactive silicon’s conductivity is also set to 5 × 10^6^ S/m. Figure 3a calculates the transmission spectra of *x*-polarized waves propagating in different directions. Two transmission resonances are observed at 2.72 THz (labeled A) and 3.84 THz (labeled B) under forward *x*-polarized wave incidence. By contrast, the transmission for the backward direction illumination is nearly zero. To further investigate the polarization-dependent transmission characteristics, Figure 3b shows the cross-polarization transmission amplitudes *T_yx_*, *T_xy_* and co-polarization transmission amplitudes *T_xx_*, *T_yy_*. The results indicate that there is a significant difference between *T_yx_* and *T_xy_*, while the co-polarized transmission amplitudes *T_xx_* and *T_yy_* are both nearly zero. Notably, *T_yx_* is close to 0.98 at 2.72 THz and 3.84 THz, indicating that the incident *x*-polarized wave is almost completely converted to *y*-polarized wave by the metamaterial. Based on the analysis of the theoretical formulas in Part II, the AT effects of *x*-polarized and *y*-polarized waves are calculated in Figure 3c. Due to the designed nanostructure corresponding well with the Lorentz reciprocity theorem, the proposed metamaterial can realize the AT effect for *y*-polarized waves of backward illumination. Meanwhile, the AT parameter for the *x*-polarized wave is equal in magnitude but opposite in sign to that of the *y*-polarized wave, that is ${∆}_{lin}^{x}=-{∆}_{lin}^{y}$. The result shows that the metamaterial can realize the dual-band nearly perfect AT effect at 2.72 THz and 3.84 THz, and the AT value is close to 0.98. Figure 3d shows the polarization conversion ratios (PCR) for *x*- and *y*-polarized waves under forward incidence. The PCR for *x*-polarization (PCR*_x_*) reaches up to 0.99 across the 1.07–4.29 THz range, while the PCR for *y*-polarization (PCR*_y_*) remains close to zero throughout. This indicates that the proposed metamaterial achieves ultra-wideband polarization conversion, with nearly complete conversion of the incident *x*-polarized wave to *y*-polarization.

**Figure 4:**
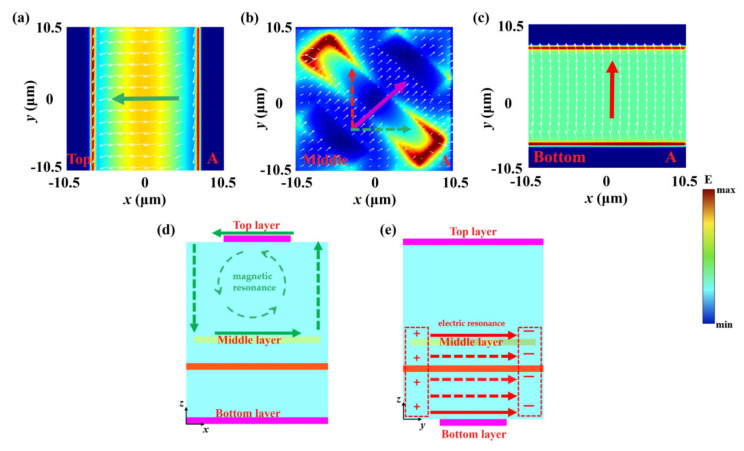
The current distributions at the resonance peak A. (**a**) The top photosensitive silicon layer, (**b**) middle Au gratings, (**c**) bottom photosensitive silicon layer, (**d**) *x*-*z* plane, (**e**) *y*-*z* plane.

**Figure 5:**
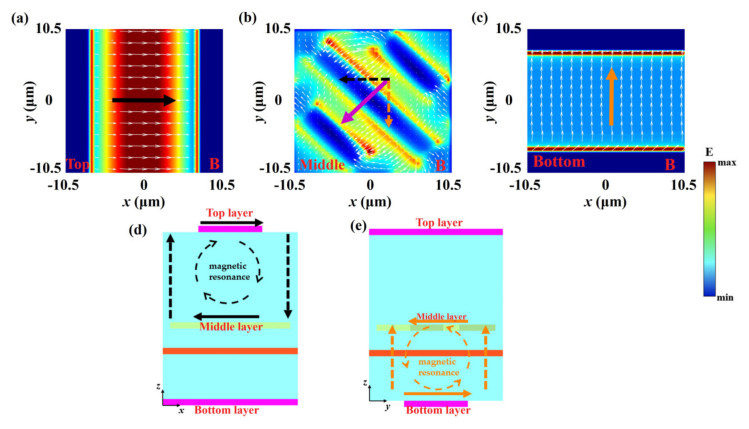
The current distributions for the resonance peak B. (**a**) The top photosensitive silicon layer, (**b**) middle Au gratings, (**c**) bottom photosensitive silicon layer, (**d**) *x*-*z* plane, (**e**) *y*-*z* plane.

To gain deeper insight into the underlying mechanisms of the AT effect and polarization conversion, Figure 4 presents the surface current distributions in photosensitive silicon layers and Au gratings at the transmission resonance peak A. As shown in Figure 4, the surface current of the middle Au gratings layer is vectorially decomposed in the *x*- and *y*-directions, and the direction of the decomposed current is shown by the dotted arrows. The current in the *x*-direction of the Au grating layer is anti-parallel to the current on the top photosensitive silicon layer. Therefore, the first-order magnetic resonance occurs in the dielectric space between the two layers, and the magnetic dipoles produce the single loop current. The current in the *y*-direction of the Au gratings layer is parallel to the current on the bottom photosensitive silicon layer, corresponding to the first-order electric resonance. The electric dipoles produce the directional current. Thus, both electric and magnetic resonances are simultaneously excited at resonance peak A. Similarly, the surface current of the Au gratings is vectorially decomposed at the resonant transmission peak B as shown in Figure 5. The current in the *x*-direction is anti-parallel to the current on the top photosensitive silicon layer while the current in the *y*-direction is also anti-parallel to that on the bottom photosensitive silicon layer. Therefore, the magnetic resonance is generated between the Au gratings and photosensitive silicon layers. In summary, the synergistic interplay of magnetic and electric resonances drives the dual-band AT effect and enables broadband polarization conversion in the transmissive regime.

**Figure 6:**
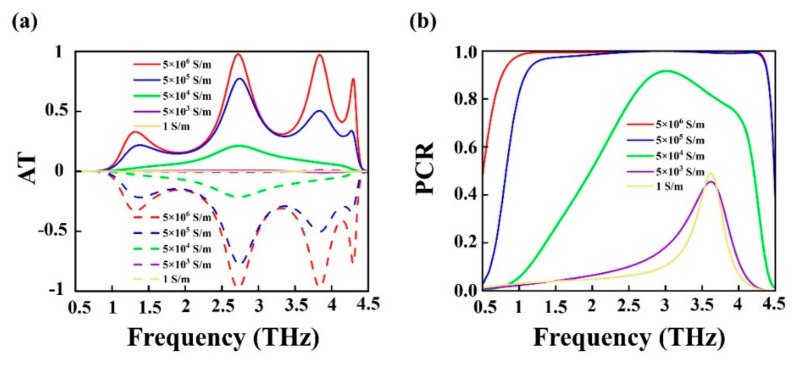
(**a**) AT and (**b**) PCR spectrum of photosensitive silicon at different conductivities. The solid line in (**a**) represents ${∆}_{lin}^{x}$, the dashed line in (**a**) represents ${∆}_{lin}^{y}$. (**b**) represents the PCR of the *x*-polarized wave propagating from the forward direction.

To explore the dynamic control of the metamaterial’s transmissivity via photosensitive silicon, we analyze the evolution of AT and PCR spectra as a function of the silicon’s tunable conductivity. As illustrated in Figure 6a, the transmittance of the dual-band AT decreases gradually as the conductivity of photosensitive silicon decreases from 5 × 10^6^ S/m to 1 S/m. Specifically, the AT value is reduced to 0.78 at 2.75 THz when the conductivity of photosensitive silicon is 5 × 10^5^ S/m. When the conductivity of photosensitive silicon is tuned to 5 × 10^4^ S/m, the AT effect is weakened significantly to only 0.21. The state of AT effect it these cases is labeled as “ON”. When the conductivity is 5 × 10^3^ S/m or less, the metamaterial is unable to exhibit the AT effect and presents an “OFF” state. As a result, the metamaterial can realize the switch of the AT effect by adjusting the conductivity of photosensitive silicon. Figure 6b shows how the PCR changes with conductivity of photosensitive silicon. One can see that the broadband PCR diminishes gradually with the reduction in conductivity. When the conductivity of photosensitive silicon drops to 1 S/m, the PCR spectra transitions from broadband to the narrowband and the PCR value decreases below 0.5. These results demonstrate that the proposed metamaterial exhibits actively tunable optical properties.

**Figure 7:**
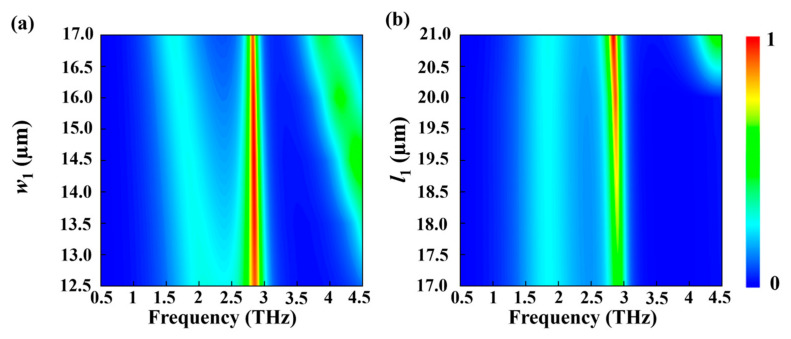
Absorption spectra as a function of frequency and structural parameters (**a**) *w*_1_ and (**b**) *l*_1_.

Figure 7 illustrates the influence of structural parameters on the absorption performance of the metamaterial when VO_2_ is in the metallic state. As shown, the absorption shows weak sensitivity to variations in *w*_1_, indicating that the width of the photosensitive silicon strip has a relatively minor effect on the impedance-matching condition. In contrast, the absorption is highly sensitive to changes in *l*_1_, which primarily governs the resonance condition of the structure. When *l*_1_ is reduced to 19 μm, the absorption efficiency decreases to 0.73. A further reduction of *l*_1_ below 18 μm significantly disrupts the resonance, causing the absorption peak to drop to approximately 0.56. This behavior can be attributed to the deviation from optimal resonance conditions, which weakens the coupling between the incident electromagnetic wave and the metamaterial structure, thereby reducing energy dissipation through ohmic loss and impedance matching.

**Figure 8:**
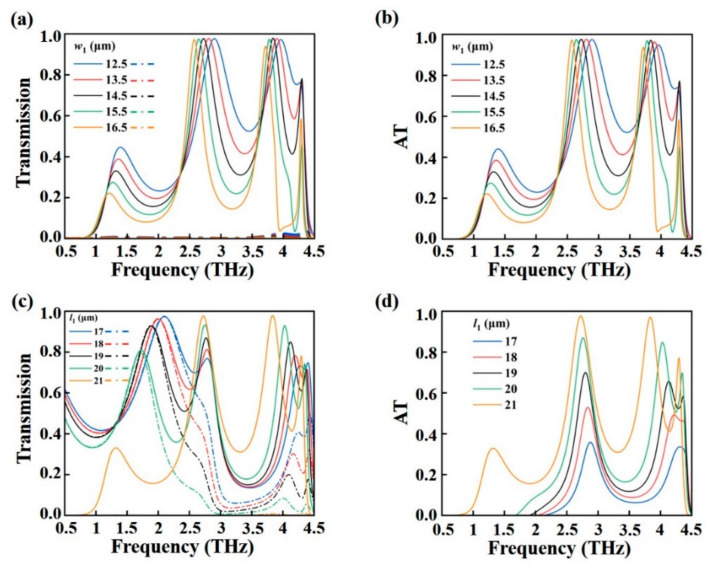
The influences of structure parameters *w*_1_ and *l*_1_ on (**a**,**c**) transmission of forward (solid lines) and backward (dashed lines) illuminations, and (**b**,**d**) AT spectra.

Figure 8 investigates the influences of structural parameters on AT effect when VO_2_ is in its insulating state. As shown in Figure 8a, the forward transmission spectrum exhibits a red shift with the increasing values of *w*_1_, while the backward transmission still tends to zero as shown in Figure 8a. In the plot, the solid lines represent the *x*-polarized wave illuminating from the forward direction, whereas the dashed lines correspond to the *x*-polarized wave propagating from the backward direction. Consequently, the AT effect shows a red shift as *w*_1_ increases from 12.5 μm to 16.5 μm in 1 μm increments, as illustrated in Figure 8b. This behavior arises because a wider *w*_1_ increases the effective optical path length and alters the resonance condition of the structure, thereby reducing the resonance frequency. Figure 8c further presents the influence of varying *l*_1_ on the transmission spectrum. One can see that there exist blue shifts for both forward and backward transmission spectrum with the decrease of *l*_1_. Figure 8d calculates the AT spectra of the proposed structure with the decrease of *l*_1_ from 21 μm to 17 μm in steps of 1 μm. It is clearly seen from Figure 8d that the AT values gradually decrease and have a slight blue shift. This can be attributed to the weakening of resonant coupling between electric and magnetic modes as the photosensitive silicon strip becomes shorter, thereby degrading the asymmetry in transmission. The above discussion demonstrates that the asymmetric transmission effect in the proposed metamaterial can be effectively tuned by tailoring structural parameters such as *w*_1_ and *l*_1_, offering a versatile approach for optimizing device performance across specific frequency bands.

To further highlight the novelty of this work, Table 1 provides a comparison with previously reported metamaterials. The table summarizes key parameters, including operating frequency range, functionality, and the functional materials employed in each design. As shown in Table 1, although some prior works have achieved the integration of perfect absorption, polarization conversion, and asymmetric transmission, their performance remains limited by insufficient efficiency and narrow modulation depth. In contrast, our proposed metamaterial exhibits superior multifunctional performance. When VO_2_ is in its metallic state, the structure achieves selective perfect absorption with near-unity absorptivity (>0.99). In the insulating state, it enables dual-band asymmetric transmission with a peak AT ratio of 0.98, while maintaining a polarization conversion ratio exceeding 0.99 over a broad bandwidth, which represents a performance metric unattained in previously reported works. Moreover, unlike earlier approaches that modulate metamaterial behavior by tuning the conductivity of VO_2_ or other materials such as graphene, our design enables full ON/OFF switching of the AT effect by varying the conductivity of photosensitive silicon, thus offering enhanced modulation depth and dynamic reconfigurability.

Finally, it is important to note that while our design leverages established nanofabrication techniques (e.g., plasma-enhanced chemical vapor deposition, pulsed laser deposition, and ion beam milling), we recognize the stringent alignment requirements for multilayer integration. To address this challenge, one can employ the stepper lithography with <5% critical dimension variation, ensuring sub-100 nm precision. This approach aligns with recent advancements in high-resolution patterning for metamaterials. Moreover, VO_2_’s thermal hysteresis (typically spanning 68–72 °C) can be mitigated through tungsten doping to lower and stabilize the transition temperature, or by incorporating local microheaters to enable precise and dynamic phase control. In addition, to extend the operational frequency range, efficiency, and robustness, future design improvements could integrate multi-resonance structures (e.g., fractal geometries or hybrid active materials) to broaden bandwidth and enable dynamic tunability. Efficiency gains may arise from doped semiconductors, while advanced fabrication techniques (stepper lithography, post-etch tuning) and thermal management strategies (microheaters, W-doped VO_2_) could address interfacial stress and thermal instability.

## 4. Conclusions

In summary, a multifunctional terahertz metamaterial based on VO_2_ and photosensitive silicon is proposed, in which perfect absorption and broadband perfect polarization conversion, accompanied by dual-band AT effect can be switched by utilizing the phase transition characteristics of VO_2_. Simulations reveal that when VO_2_ is in its metallic state, the metamaterial functions as a polarization-selective THz absorber, exhibiting near-perfect *x*-polarized absorption at 2.84 THz, while retaining a negligible absorption for *y*-polarized waves. When VO_2_ is in the insulating state, the PCR is above 99% at the band of 1.07–4.29 THz, accompanied by dual-band AT effect at frequencies of 2.72 THz and 3.84 THz. Moreover, the polarization conversion performance and the OFF/ON state of AT effect can be tuned by changing the intensities of the pump light acting on the photosensitive silicon. Based on the above phenomenon, this metamaterial design may enable novel multifunctional THz photonic platforms.

## Figures and Tables

**Table 1:** Comparisons of the proposed metamaterial with previous similar works.

Refs.	Operating Frequency Range	Functionality	Active Material
[20]	5.2–7.1 THz	Absorption ~0.95, AT effect ~0.72 and polarization conversion ~0.95	VO_2_
[21]	1.25–2.5 THz	Single-band selective absorption ~0.99, polarization conversion and AT ~0.9	VO_2_
[29]	3.0–6.5 THz	Switch between single-band AT ~0.58 and dual-band AT ~0.86, polarization conversion ~0.99	VO_2_
[39]	7.5–10.7 THz	AT ~0.34, linear to linear and linear to circular polarization conversion ~0.99	Graphene and VO_2_
[48]	1.15–1.88 THz	AT ~0.73, polarization conversion ~0.8, Absorption ~0.9	VO_2_
[49]	0.6–1.0 THz	Single-band AT ~0.95, polarization conversion ~0.99 and linear dichroism ~0.9	VO_2_
[50]	3.56–7.2 THz	AT effect ~0.75, polarization conversion ~0.95 and absorption ~0.95	VO_2_
This work	0.5–4.5 THz	Single-band selective absorption ~0.99, broadband polarization conversion ~0.99 and dual-band AT ~0.98	VO_2_ and photosensitive silicon

## Data Availability

The data are available from the corresponding author upon reasonable request.
